# Risk for Epidemics after Natural Disasters

**DOI:** 10.3201/eid1305.070080

**Published:** 2007-05

**Authors:** Rémy Michel, Jean-Paul Demoncheaux, Jean-Paul Boutin, Dominique Baudon

**Affiliations:** * Institut de Médecine Tropicale du Service de Santé des Armées, Marseille, France; † Direction Régionale du Service de Santé des Armées, Lyon, France

**Keywords:** communicable diseases, epidemics, natural disasters, letter

**To the Editor:** Myths that disaster-affected populations are at high risk for outbreaks and that dead bodies contribute to this risk are common (*1*). Conversely, some experts deny high, short-term risk after disasters (*2*).

We agree with Watson et al. (*3*) that the risk for communicable diseases transmission after natural disasters is low but real and that it is not directly related to the disasters and dead bodies, but primarily associated with the characteristics of the displaced population within the local disease ecology. This belief supports the need for rapid but accurate assessment of health status, risk, and needs, the results of which greatly influence the nature of relief activities (*4*). Key functions of relief teams are communicable diseases surveillance, early warning, and rapid response to epidemic-prone situations or outbreaks.

As an example, on October 26, 2005, after an earthquake in Pakistan, the World Health Organization asked the French military epidemiologic assessment team (1 epidemiologist and 1 veterinarian) to perform a sanitary assessment after cases of acute bloody diarrhea were reported in the camp of Tariqabad (estimated population ≈2,000), near Muzaffarabad. The assessment highlighted a lack of safe water and sanitation facilities, low routine immunization coverage, and disruption of healthcare services.

To prevent further diarrhea, we recommended improving the overall water and sanitation conditions. A medical team from a French nongovernment organization was also provided to help the 1 physician at the camp. Concurrently, we recommended a vaccination campaign as preventive strategy against diseases likely to occur in such conditions: tetanus, diphtheria, and measles. These measures were quickly implemented to reduce the overall risk, and no further unusual increases in disease incidence were noted during the following weeks. As in another outbreak documented in a camp in the Muzaffarabad area (*5*), rapid detection, response, and implementation of control measures are critical for minimizing the illness and death associated with outbreaks in these high-risk populations.
